# Supplementary material for: The histone demthylase KDM3A protects the myocardium from ischemia/reperfusion injury via promotion of ETS1 expression
Source: Commun Biol. 2022 Mar 25;5:270. doi: 10.1038/s42003-022-03225-y (PMC8956629; doi:10.1038/s42003-022-03225-y)
Supplement: Supplementary file 2 — Supplementary Information [file 42003_2022_3225_MOESM2_ESM.pdf]

Supplementary information

Supplementary Figure 1.

A CRISPR/Cas9 genome-editing technology was operated to generate KDM3A-KO (*kdm3a*$^{-/-}$) rat. (a) One single guide RNA (sgRNA) flanked exon 5 of KDM3A gene in rat was designed and created. (b) PCR gel electrophoresis show KDM3A products on wild type (*kdm3a*$^{+/+}$), heterozygote *kdm3a*$^{+/-}$ and homozygous *kdm3a*$^{-/-}$ hearts. (c) The sequencing chromatograms of homozygous mutants and *kdm3a*$^{+/+}$ heart revealed a 14 bases absence. (d) Western blotting was performed to validate KDM3A protein expression in *kdm3a*$^{+/+}$ and *kdm3a*$^{-/-}$ hearts.

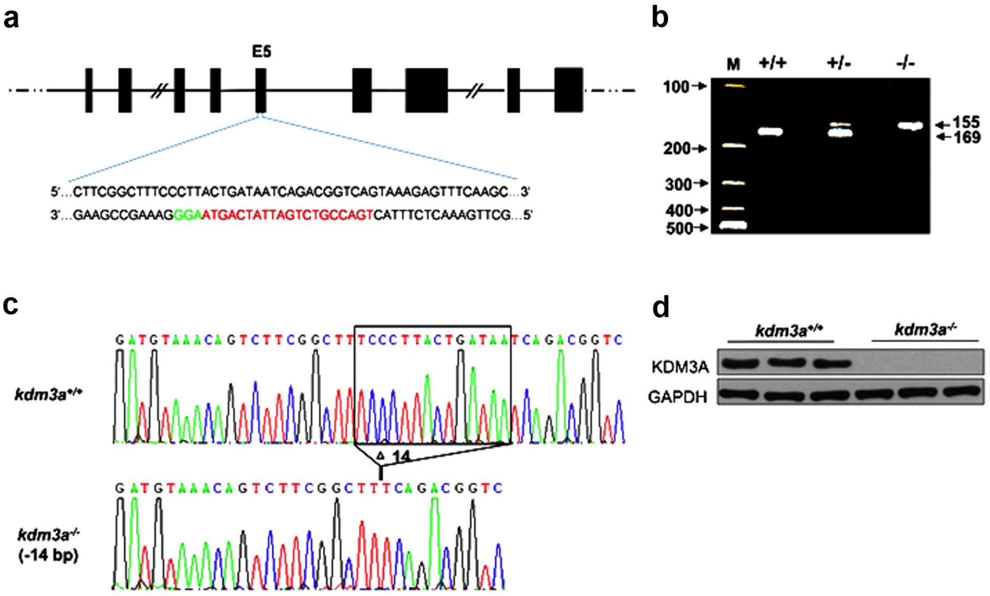

Supplementary Figure 2.

NRCMs infected with AdshKDM3A markedly decrease the expression of KDM3A while AdKDM3A evidently increased the expression of KDM3A.

a, Protein level of KDM3A in NRCMs infected with AdshKDM3A or AdKDM3A (n=6, *P<0.05 vs. AdGFP or AdshRNA). Left: representative blots; right: quantitative results.

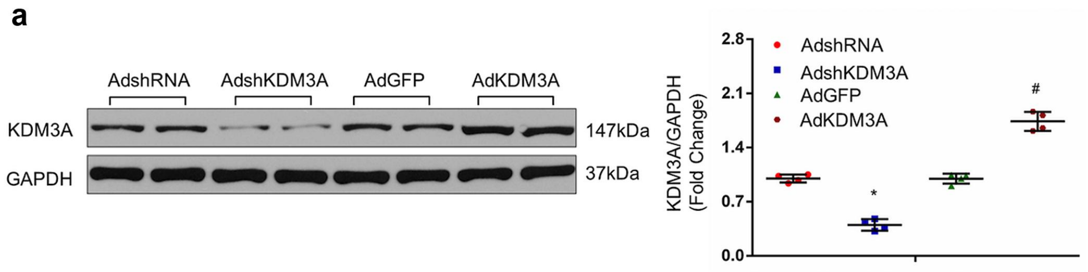

Uncropped blots

Uncropped blot corresponding to Figure 1a and Figure 1d

KDM3A

GAPDH

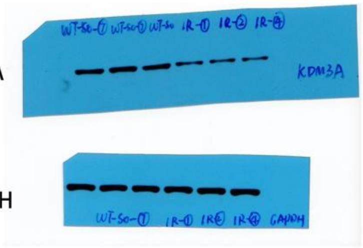

figure1a

KDM3A

GAPDH

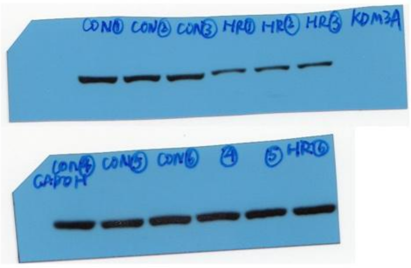

figure1d

Uncropped blot corresponding to Figure 2a.

KDM3A

GAPDH

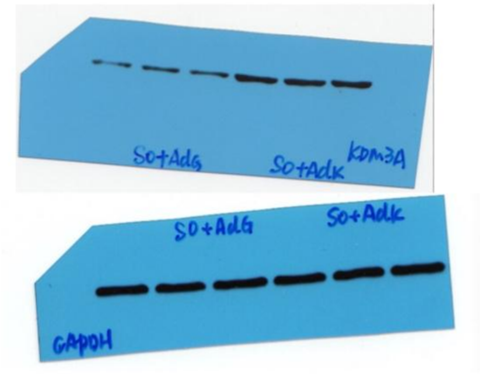

Figure2a

Uncropped blot corresponding to Figure 3e and 3f.

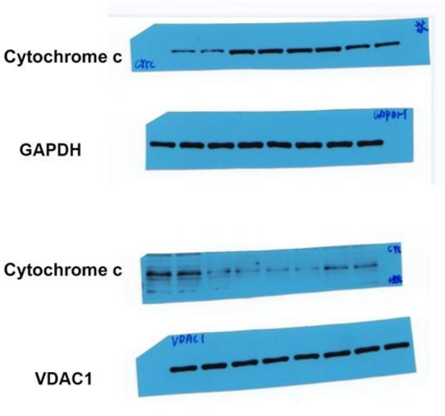

Figure3e

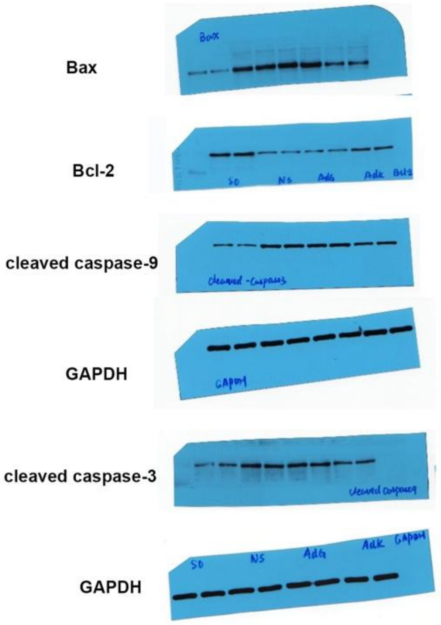

Figure3f

Uncropped blot corresponding to Figure 4e.

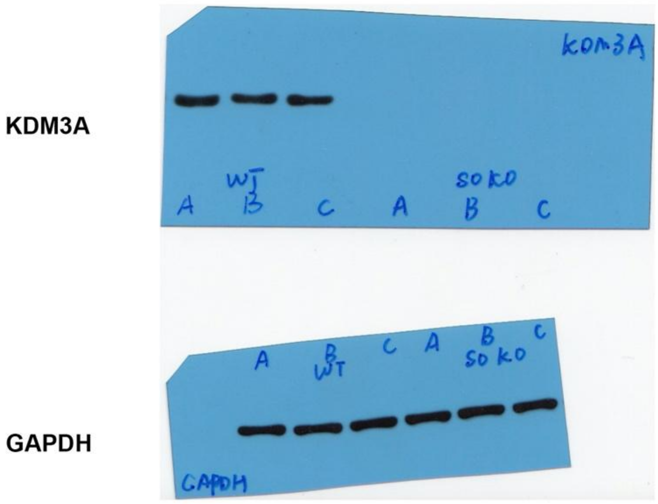

Figure 4e

Uncropped blot corresponding to Figure 5e and 5f.

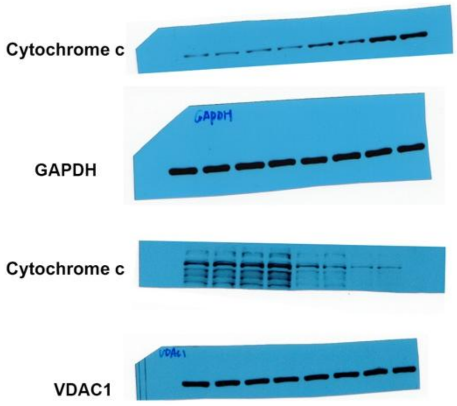

Figure5e

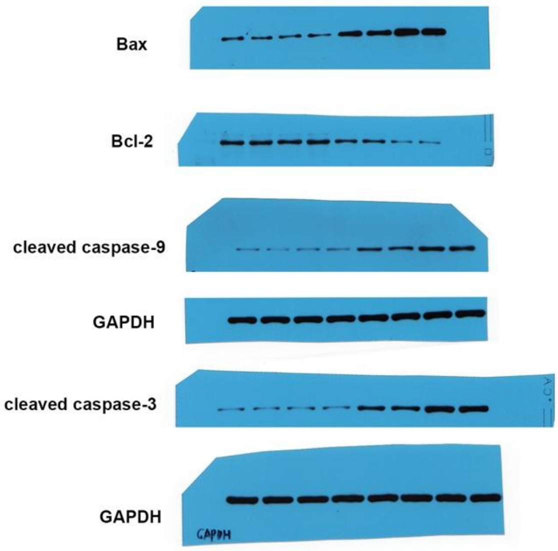

Figure5f

Uncropped blot corresponding to Figure 6g,6i,6j, and 6h.

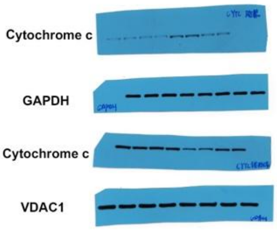

Figure6g

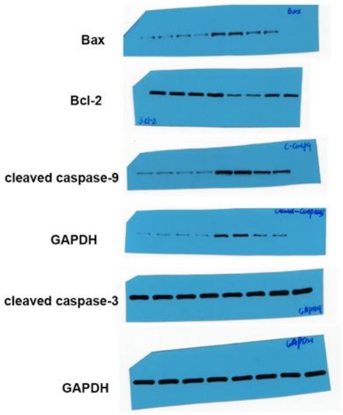

Figure6i

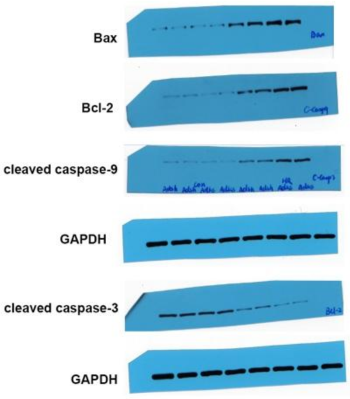

Figure6j

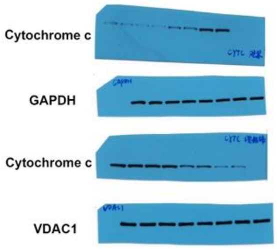

Figure6h

Uncropped blot corresponding to Figure 7b,7d,7f, and 7h.

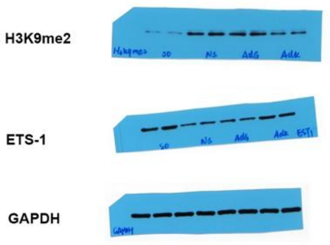

Figure7b

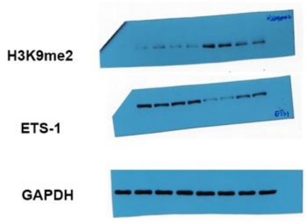

Figure7f

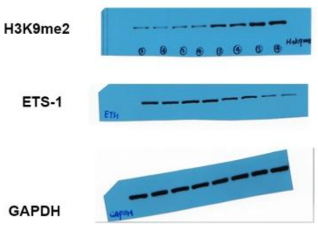

Figure7d

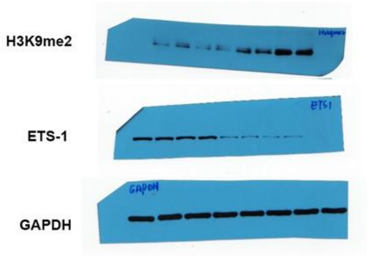

Figure7h

Uncropped blot corresponding to Figure 9a.

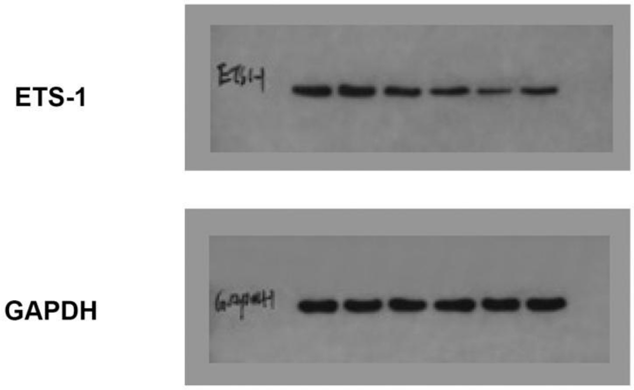

Figure9a

Uncropped blot corresponding to supplementary figure 1d and figure 2a.

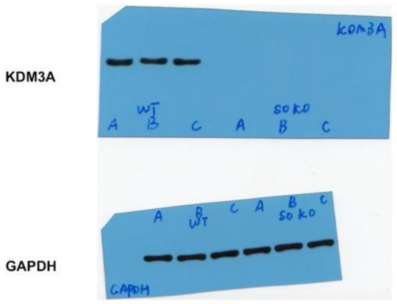

Supplementary Figure 1d

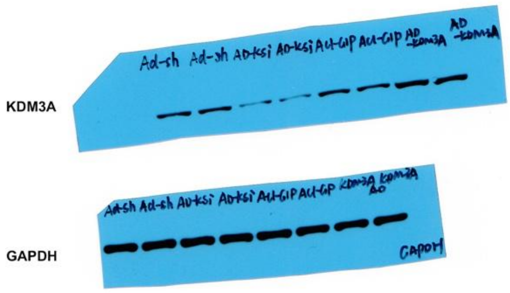

Supplementary Figure 2a

Images of the original Western Blots films

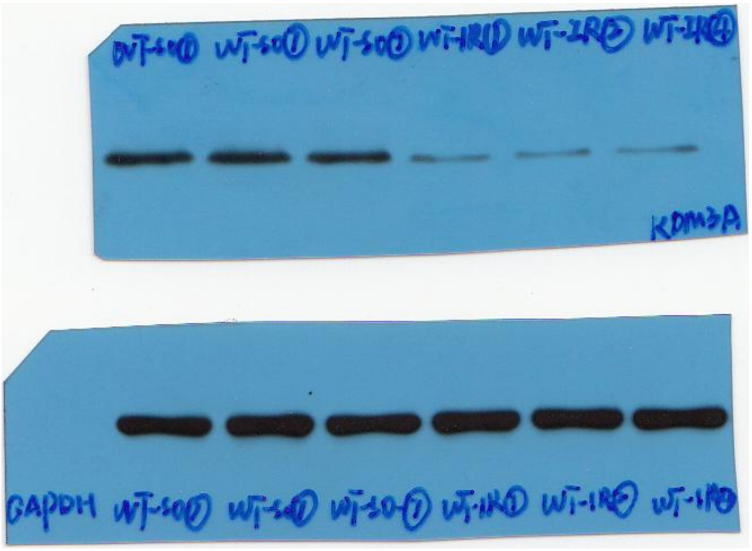

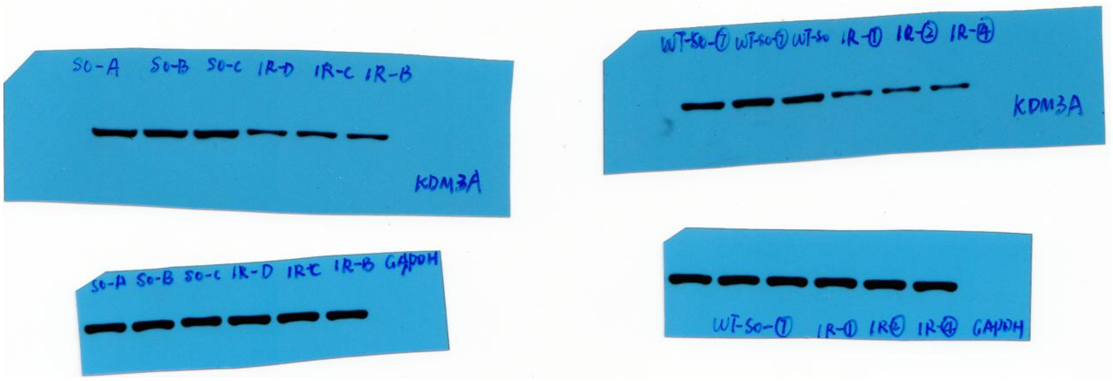

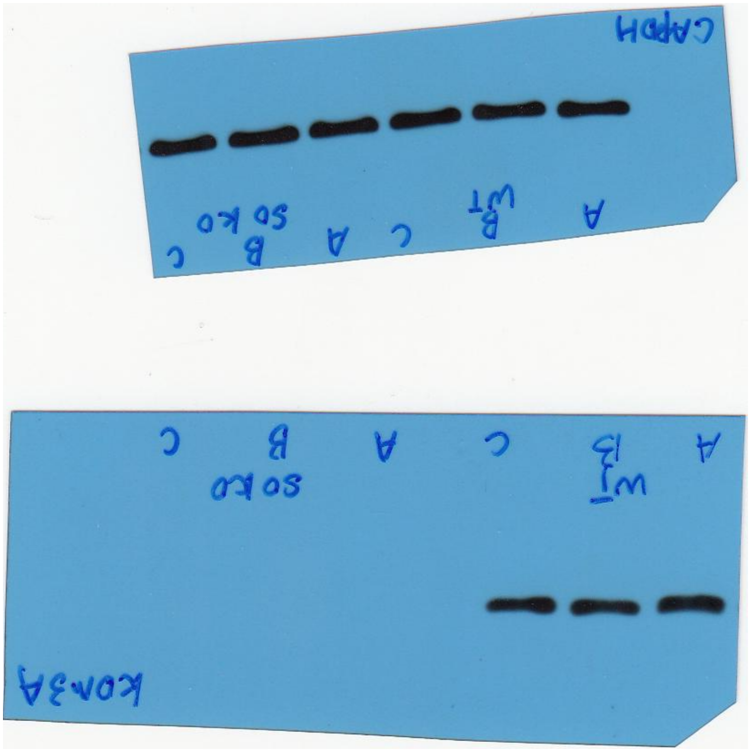

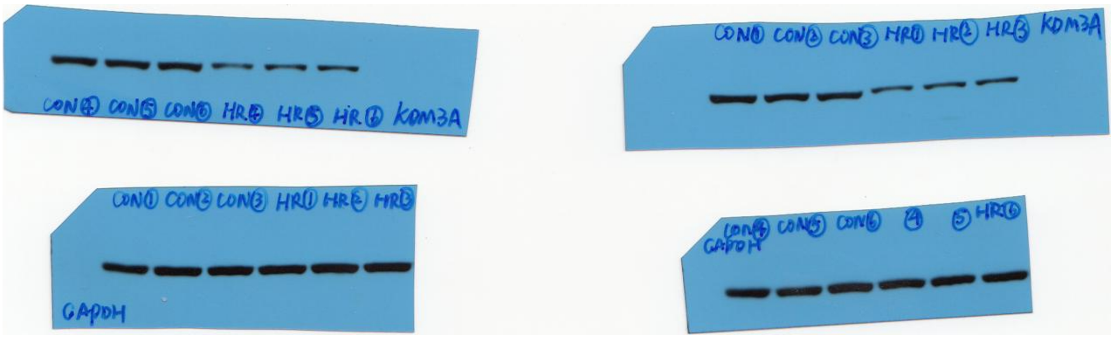
CON4 CON5 CON6 HR4 HR5 HR6 KDM3A
CON1 CON2 CON3 HR1 HR2 HR3 KDM3A
CON1 CON2 CON3 HR1 HR2 HR3
GAPDH
CON4 CON5 CON6 4 5 HR6
GAPDH

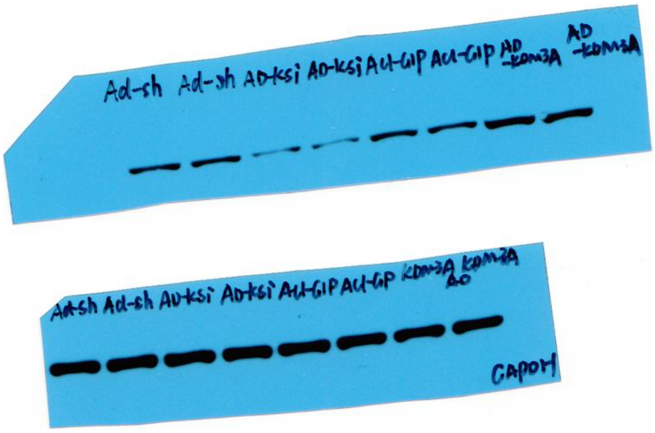
Ad-sh Ad-sh AD-ksi AD-ksi AU-GIP AU-GIP AD-KDM3A AD-KDM3A
Ad-sh Ad-sh AD-ksi AD-ksi AU-GIP AU-GIP KDM3A KDM3A AD
GAPDH

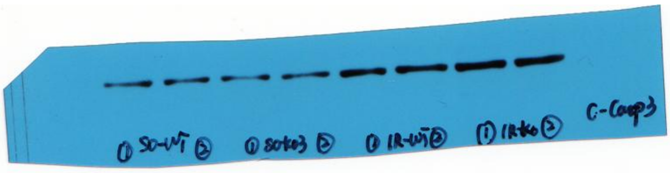
C-Casp3
① SO-WT ② ① SO-KO3 ② ① IR-WT ② ① IR-KO ②

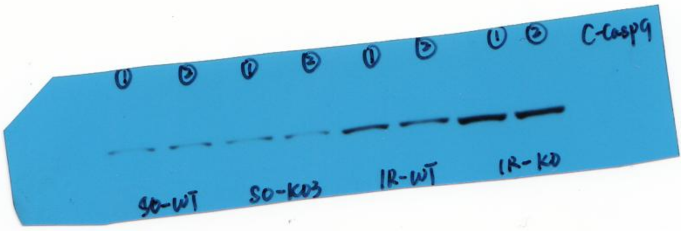
C-Casp9
① ② ① ② ① ② ① ②
SO-WT SO-KO3 IR-WT IR-KO

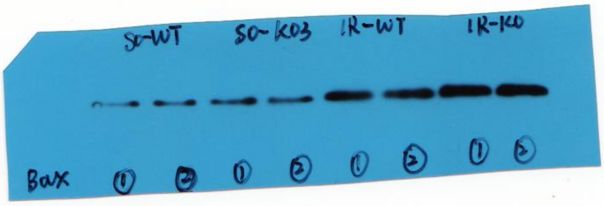
SO-WT SO-KO3 IR-WT IR-KO
Bax ① ② ① ② ① ② ① ②

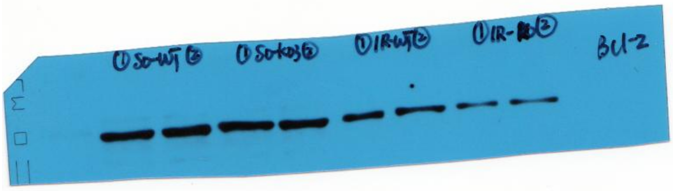
① SO-WT ② ① SO-KO3 ② ① IR-WT ② ① IR-KO ②
Bcl-2

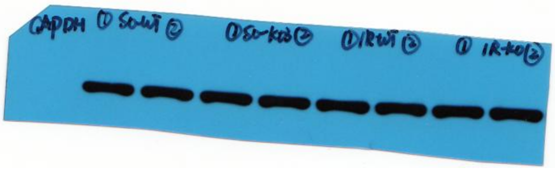
GAPDH ① SO-WT ② ① SO-KO3 ② ① IR-WT ② ① IR-KO ②

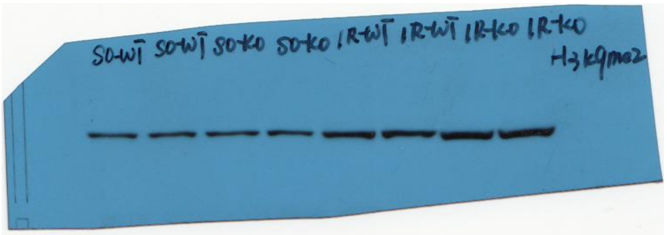
SO-WT SO-WT SO-KO SO-KO IR-WT IR-WT IR-KO IR-KO
H3k9me2

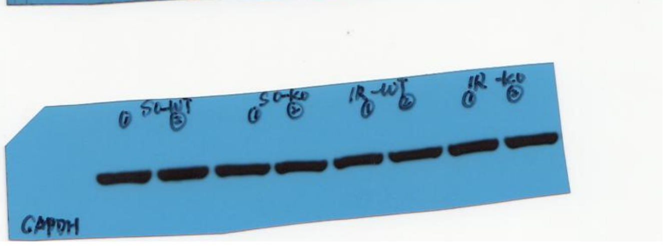
① SO-WT ② ① SO-KO ② IR-WT ① ② ① IR-KO ②
GAPDH

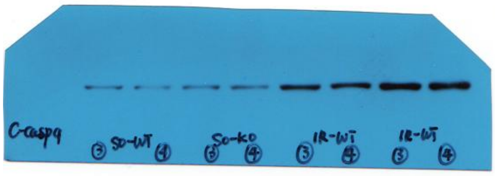
C-casp9
③ SO-WT ④
③ SO-KO ④
③ IR-WT ④
③ IR-WT ④

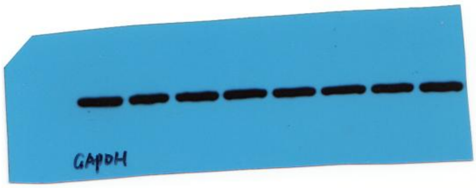
GAPDH

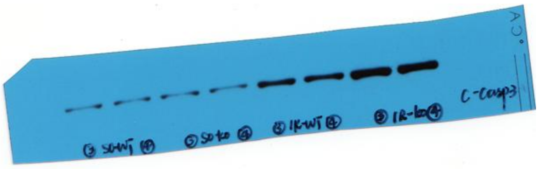
C-casp3
③ SO-WT ④ ③ SO-KO ④ ③ IR-WT ④ ③ IR-KO ④

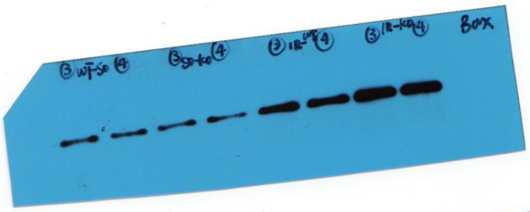
Bax
③ WT-SO ④ ③ SO-KO ④ ③ IR-WT ④ ③ IR-KO ④

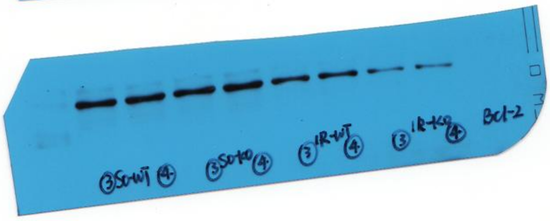
Bcl-2
③ SO-WT ④ ③ SO-KO ④ ③ IR-WT ④ ③ IR-KO ④

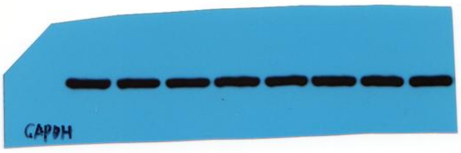
GAPDH

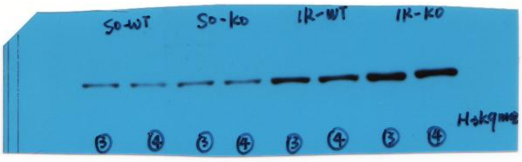
SO-WT SO-KO IR-WT IR-KO
③ ④ ③ ④ ③ ④ ③ ④
Hsk9me

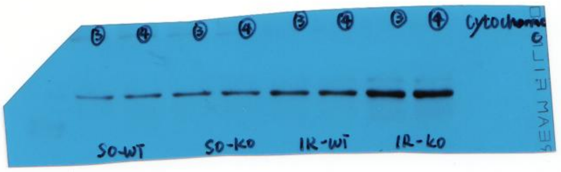
③ ④ ③ ④ ③ ④ ③ ④ Cytochrome
SO-WT SO-KO IR-WT IR-KO

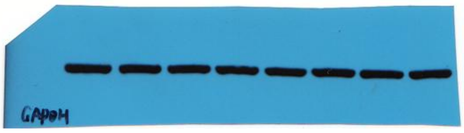
GAPDH

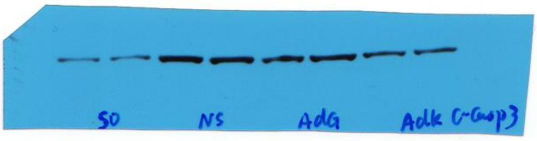
SO
NS
AdG
Adk
C-Casp3

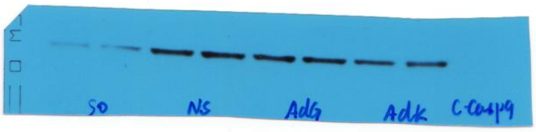
SO
NS
AdG
Adk
C-Casp9

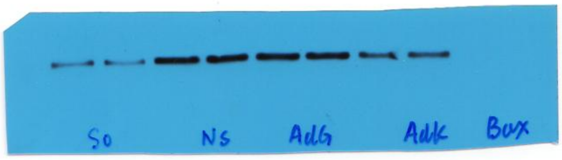
SO
NS
AdG
Adk
Bax

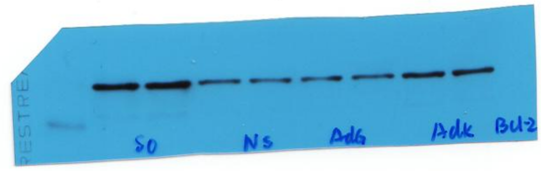
SO
NS
AdG
Adk
Bcl-2

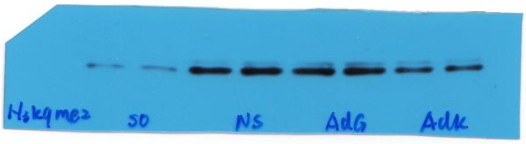
H3k9me2
SO
NS
AdG
Adk

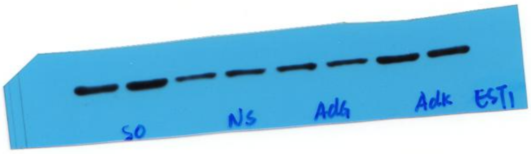
SO
NS
AdG
Adk
EST1

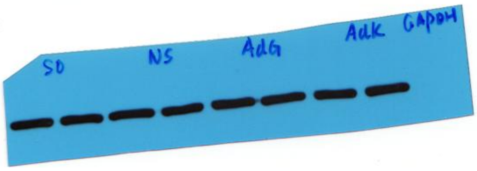
SO
NS
AdG
Adk
GAPDH

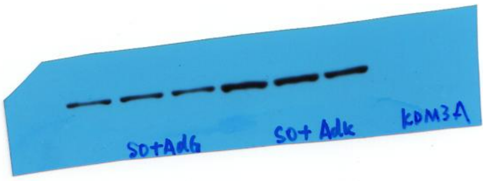
SO+AdG
SO+Adk
KDM3A

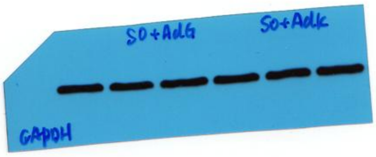
SO+AdG
SO+Adk
GAPDH

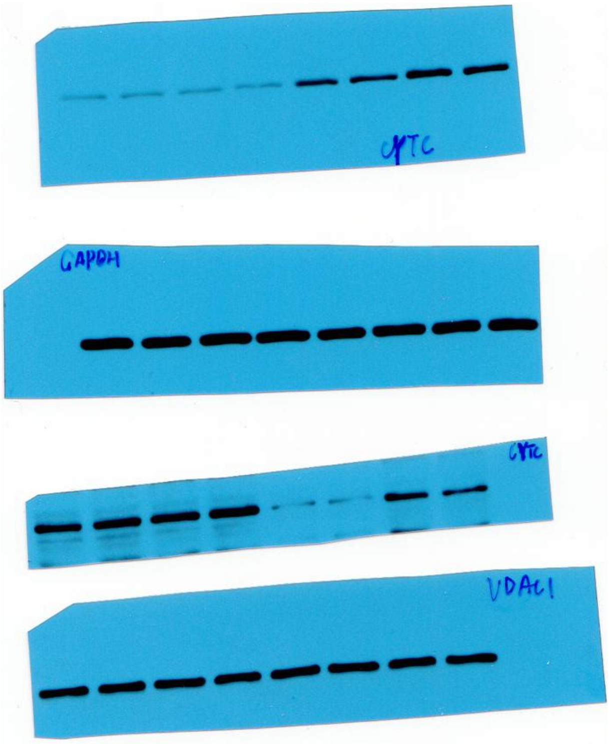
CYTC
GAPDH
CYTC
VDAC1

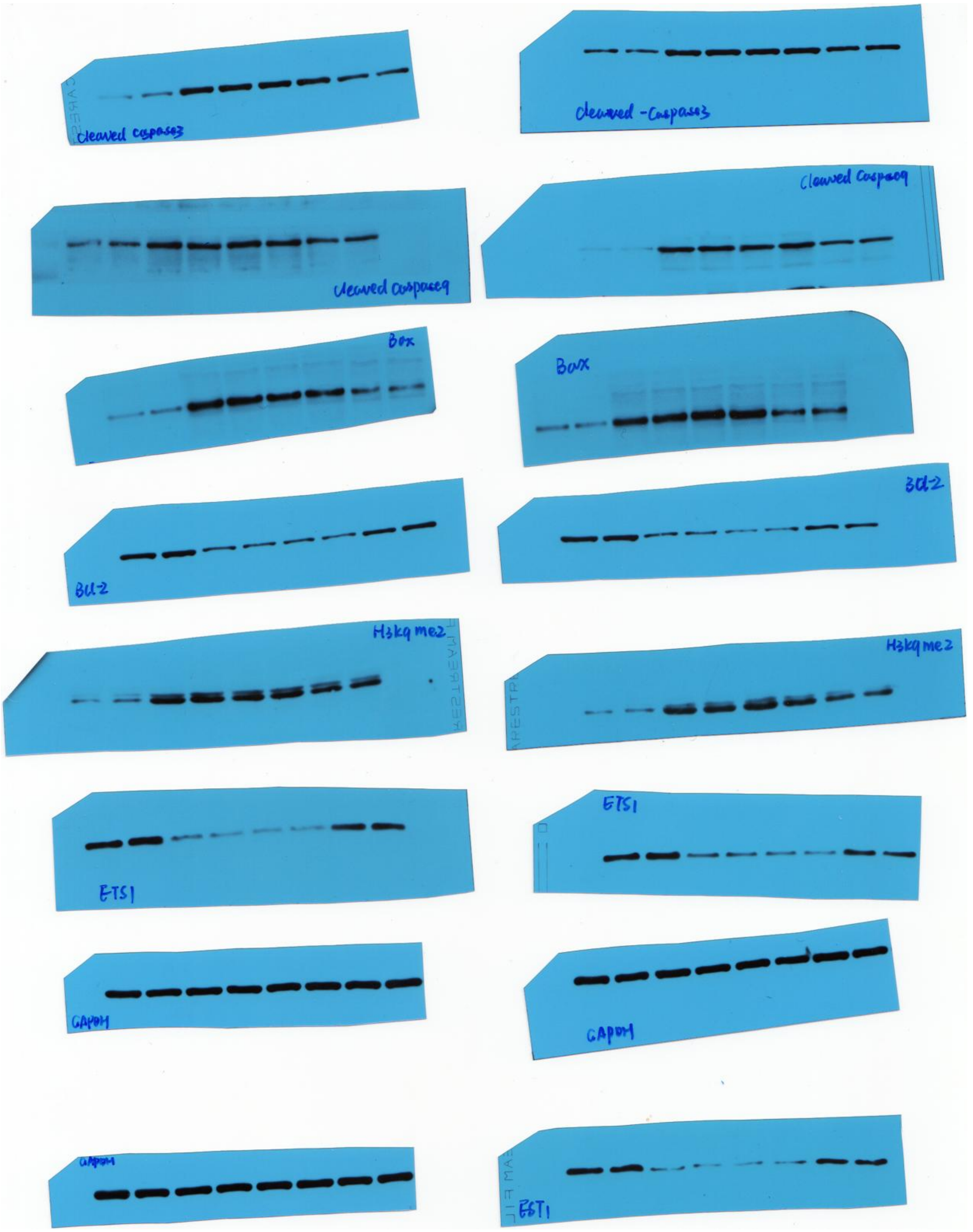
cleaved caspase3
cleaved -Caspase3
cleaved caspase9
Cleaved Caspase9
Bax
Bax
Bcl-2
Bcl-2
H3k9 me2
H3k9 me2
ETS1
ETS1
GAPDH
GAPDH
GAPDH
ESTI

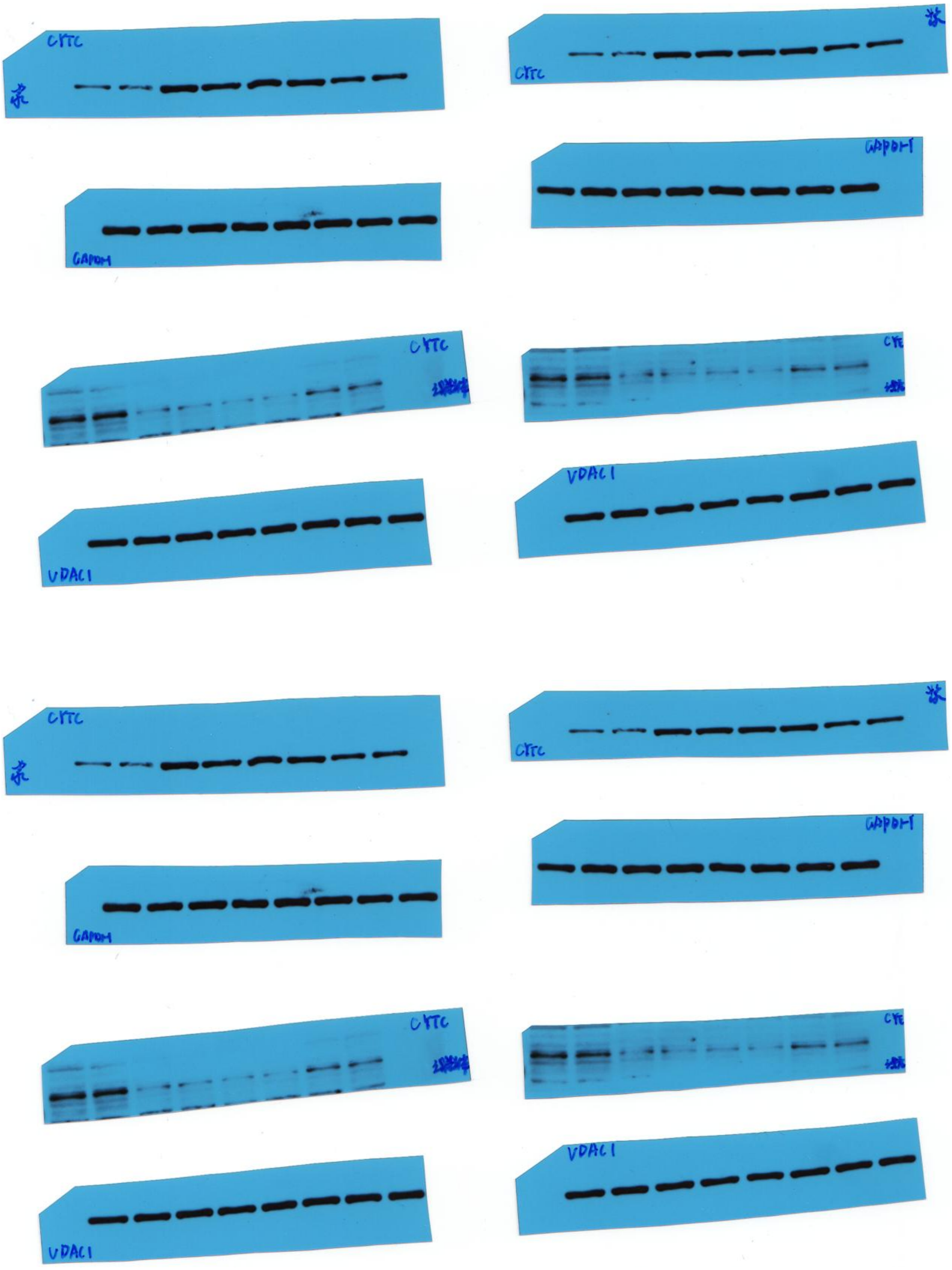
CYTC
GAPDH
VDAC1

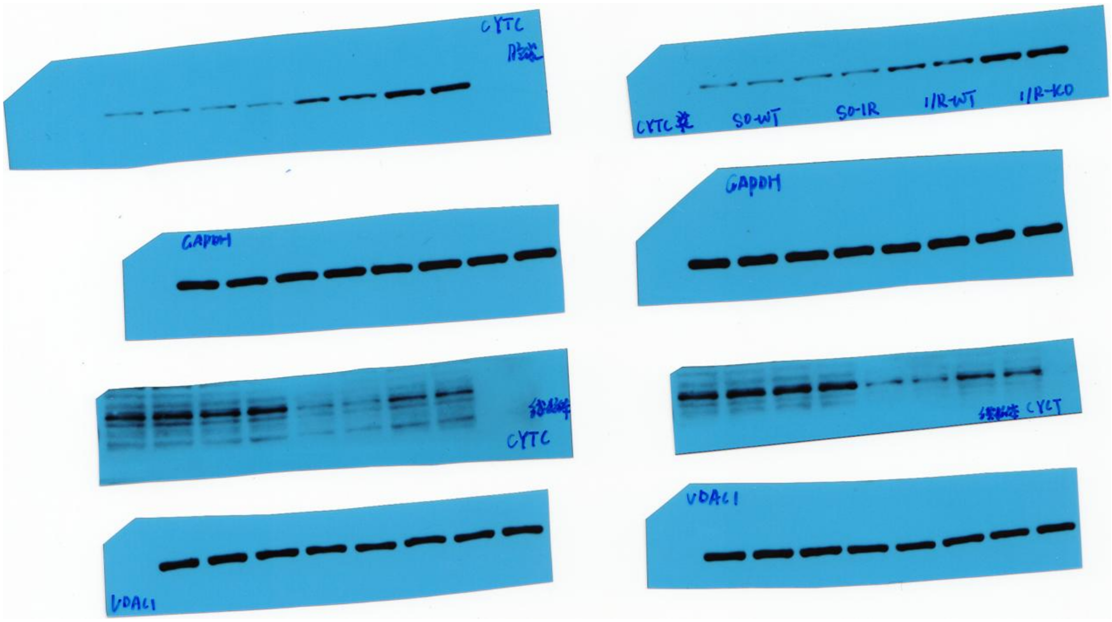
CYTC
SO-WT
SO-IR
I/R-WT
I/R-KO
GAPDH
GAPDH
CYTC
CYCT
VDAC1
VDAC1

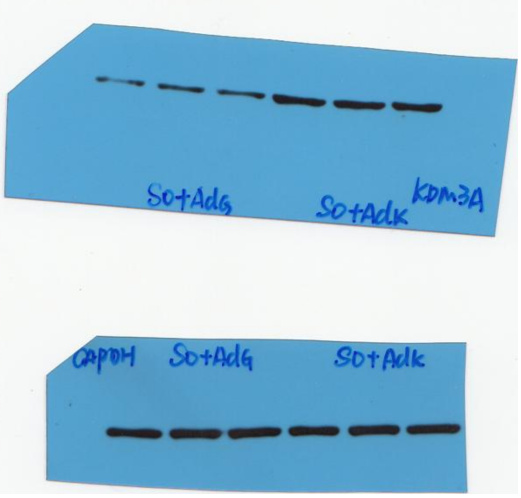
SO+AdG
SO+AdK
KDM3A
GAPDH
SO+AdG
SO+AdK

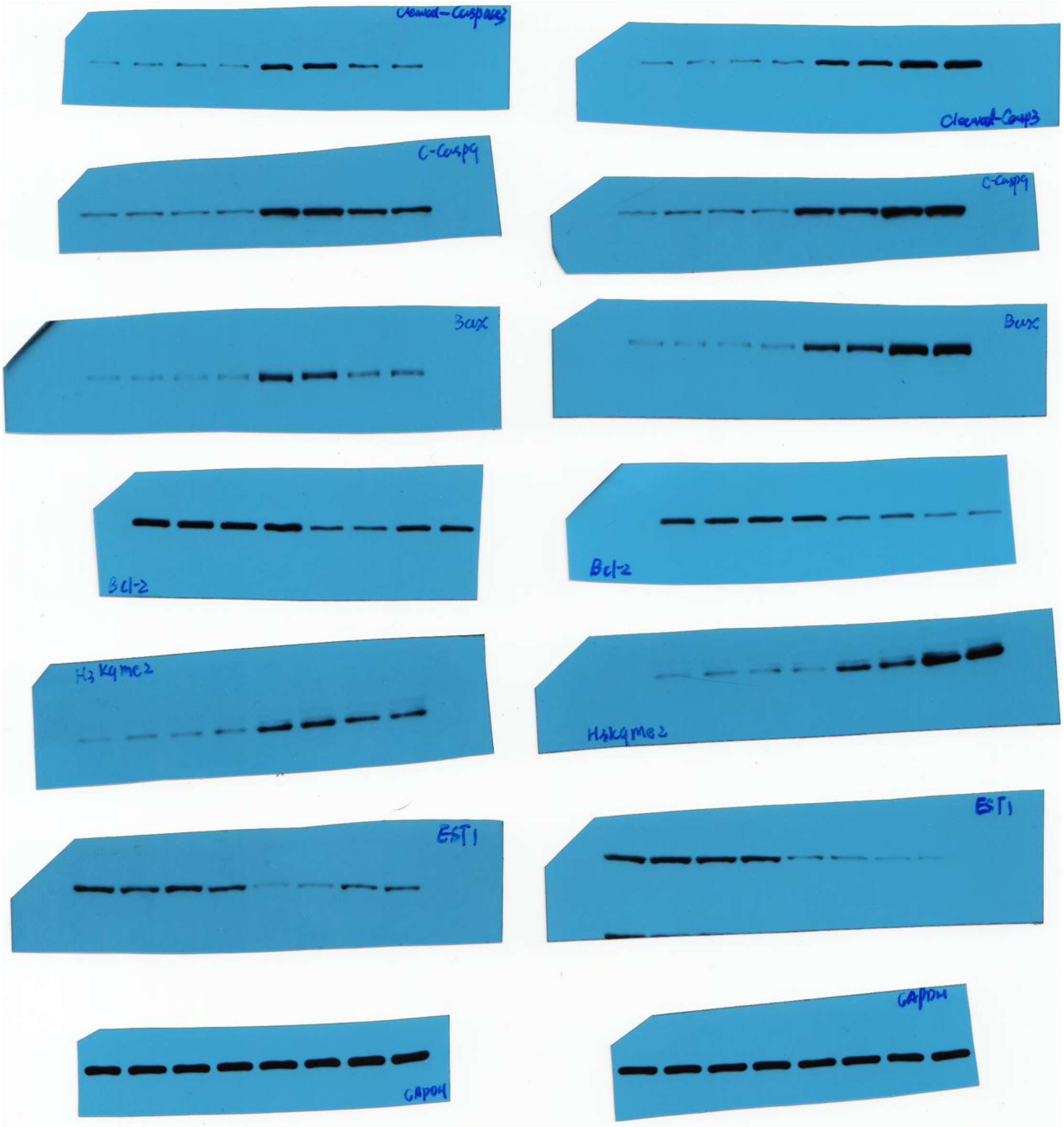
Cleaved-Caspase3
Cleaved-Casp3
C-Casp9
C-Casp9
Bax
Bax
Bcl-2
Bcl-2
H3K9me2
H3K9me2
EST1
EST1
GAPDH
GAPDH

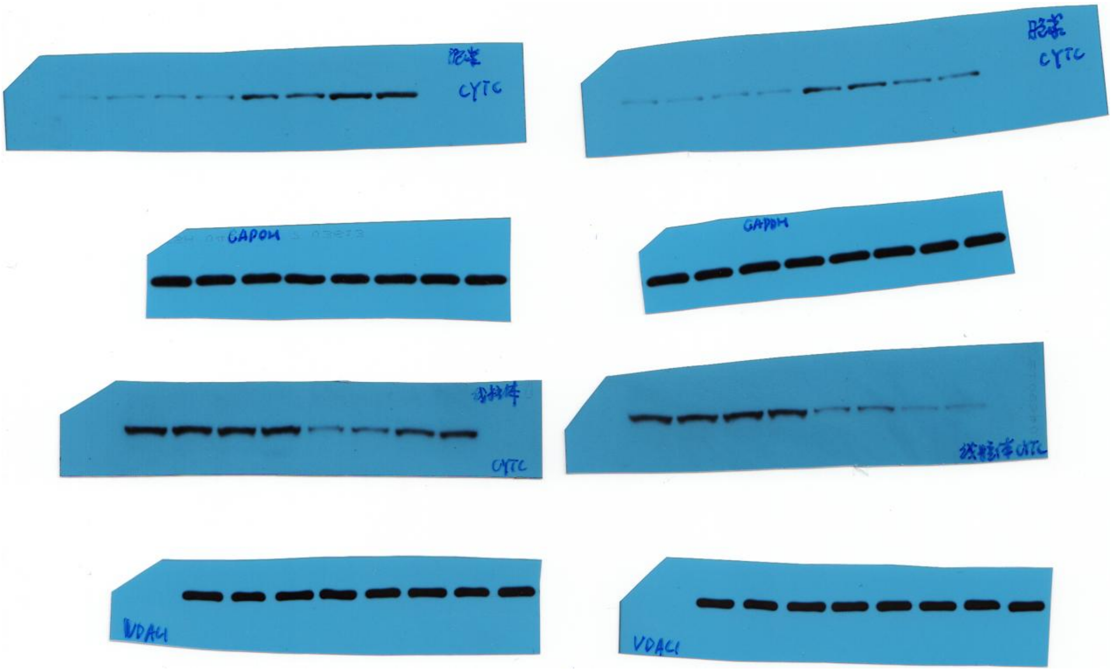
胞浆
CYTC
胞浆
CYTC
GAPDH
GAPDH
线粒体
CYTC
线粒体 CYTC
VDAC1
VDAC1

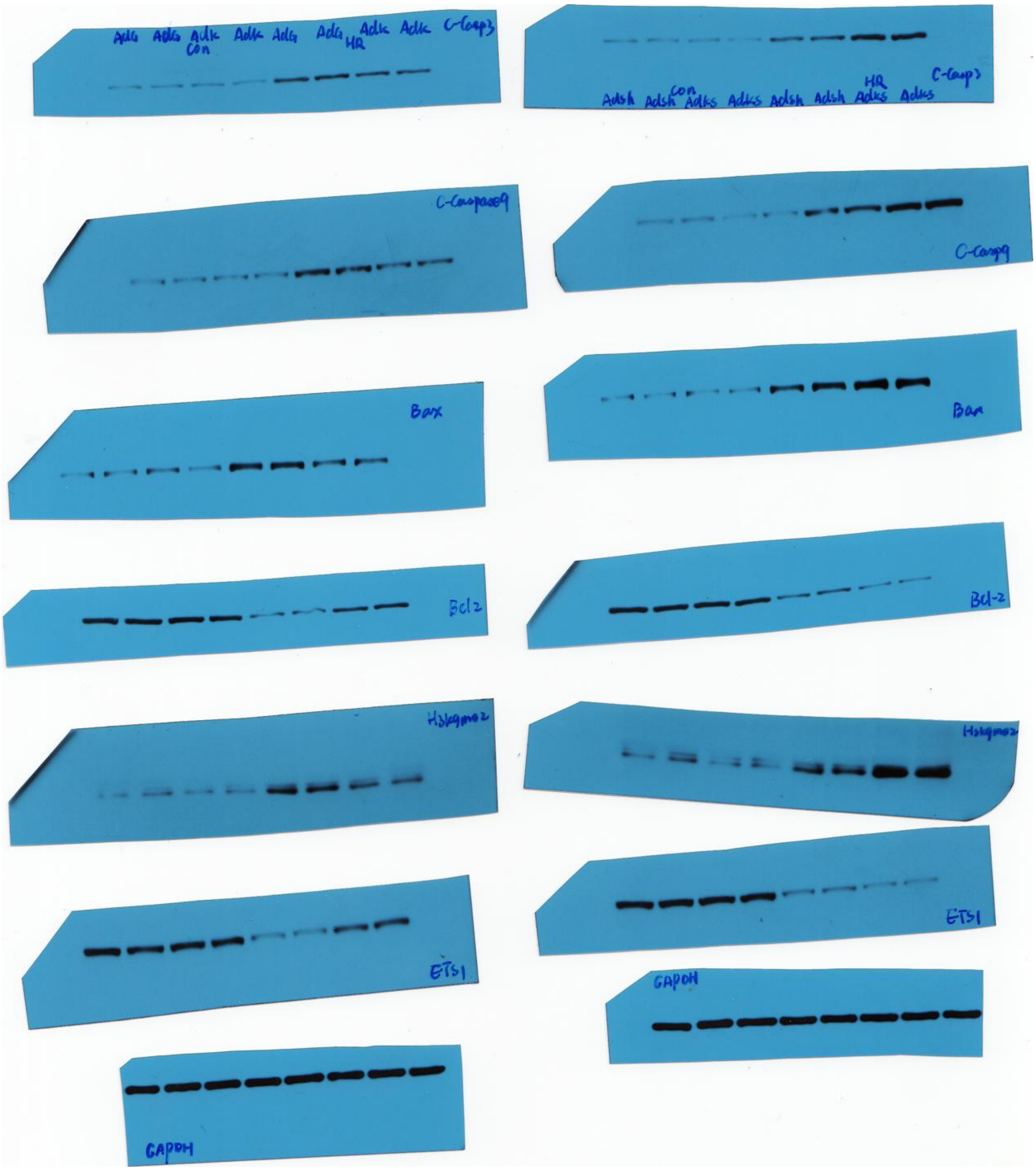

Con
HR
C-Casp3
Adsh Adsh Adks Adks Adsh Adsh Adks Adks
C-Caspase9
Bax
Bcl2
Bcl-2
ETS1
GAPDH

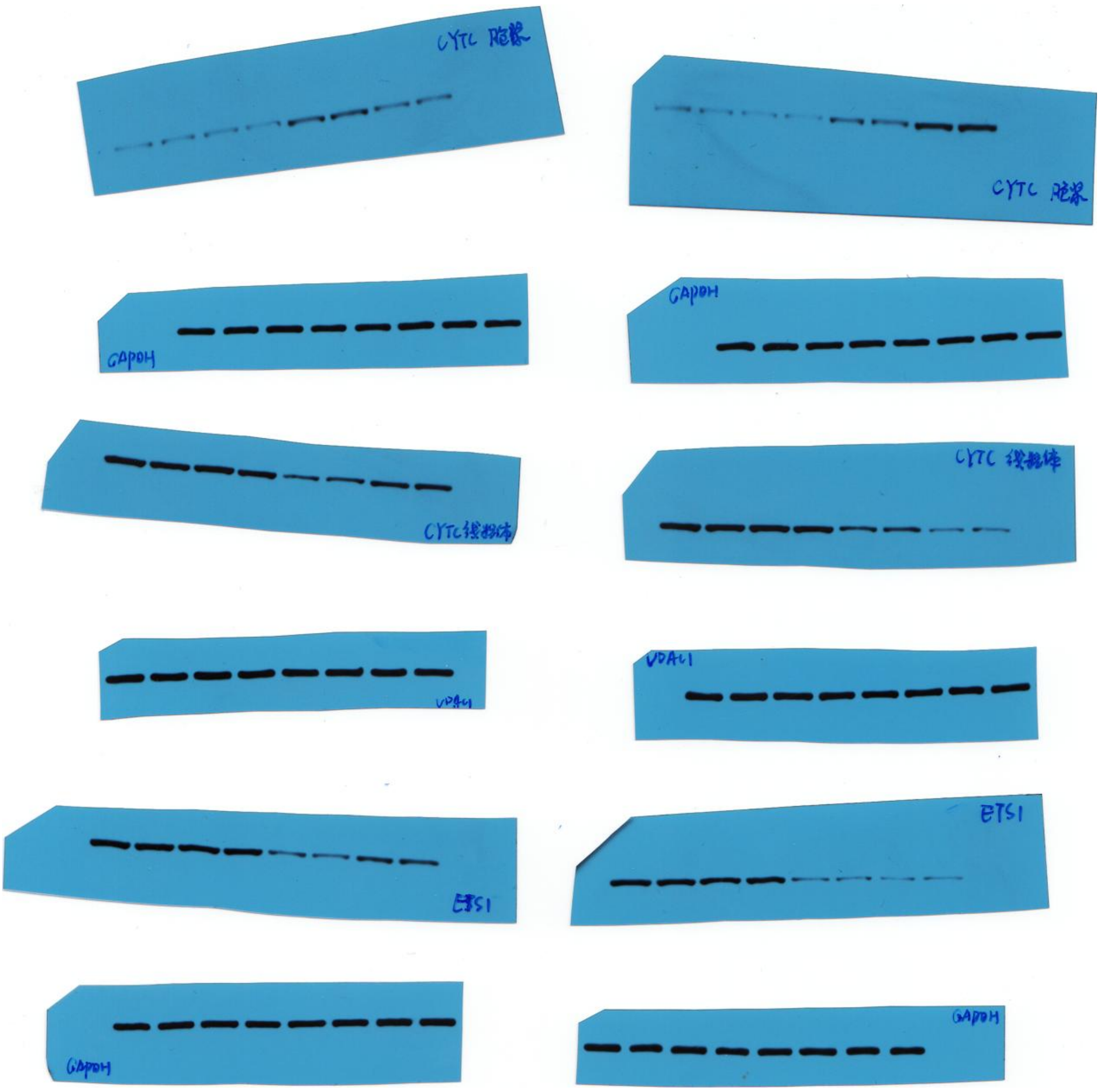
CYTC 胞浆
CYTC 胞浆
GAPDH
GAPDH
CYTC 线粒体
CYTC 线粒体
VDAC1
VDAC1
ETS1
ETS1
GAPDH
GAPDH

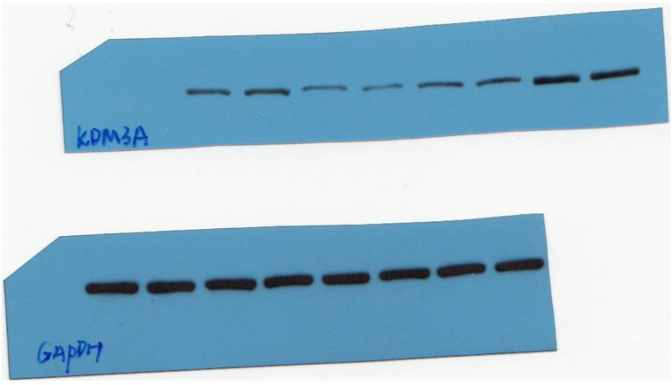
KDM3A
GAPDH

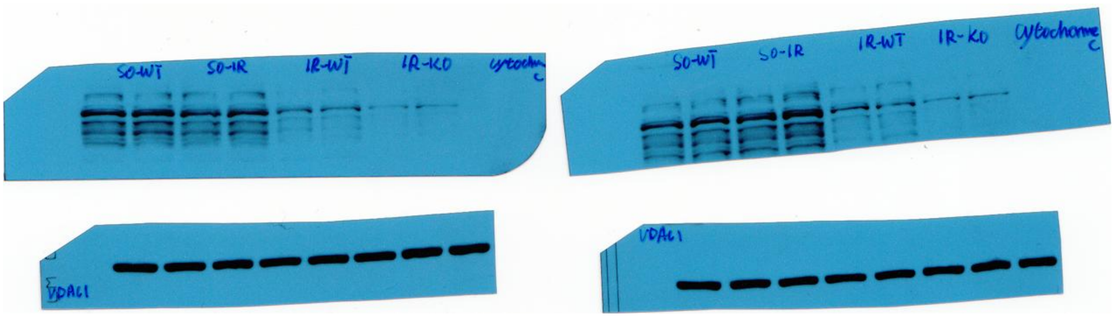
SO-WT
SO-IR
IR-WT
IR-KO
Cytochome c
VDAC1

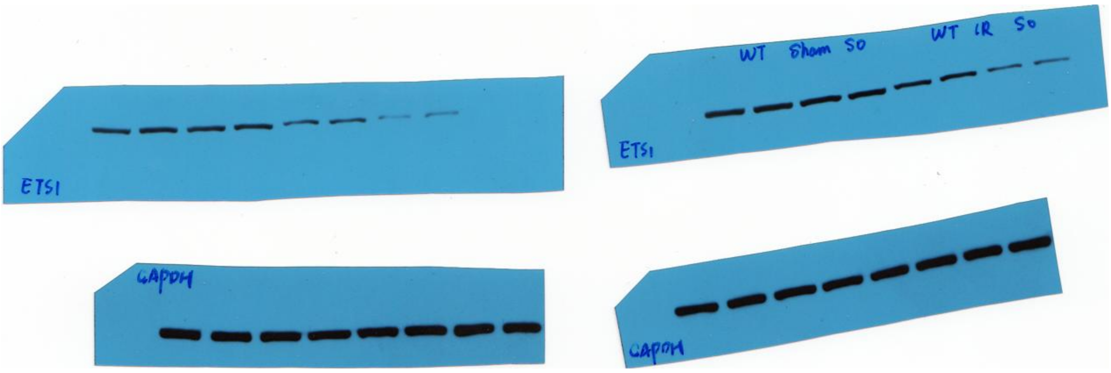
WT
Sham
SO
WT
IR
SO
ETS1
GAPDH

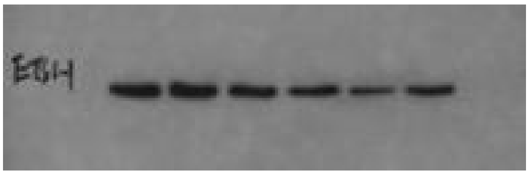
ETS1

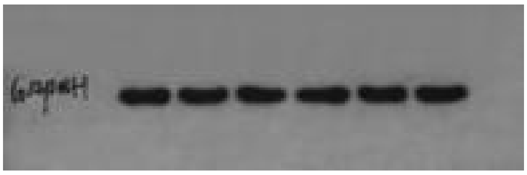
GAPDH
