# Supplementary material for: The histone demthylase KDM3A protects the myocardium from ischemia/reperfusion injury via promotion of ETS1 expression
Source: Commun Biol. 2022 Mar 25;5:270. doi: 10.1038/s42003-022-03225-y (PMC8956629; doi:10.1038/s42003-022-03225-y)
Supplement: Supplementary file 3 — Description of Additional Supplementary Files [file 42003_2022_3225_MOESM3_ESM.pdf]

Description of Additional Supplementary Files

File name: Supplementary Data 1
Description: Source data for graphs and charts.
