# Supplementary material for: The histone demthylase KDM3A protects the myocardium from ischemia/reperfusion injury via promotion of ETS1 expression
Source: Commun Biol. 2022 Mar 25;5:270. doi: 10.1038/s42003-022-03225-y (PMC8956629; doi:10.1038/s42003-022-03225-y)
Supplement: Supplementary file 5 — Reporting Summary [file 42003_2022_3225_MOESM5_ESM.pdf]

nature portfolio

Corresponding author(s): COMMSBIO-21-2408B

Last updated by author(s): Jan 10, 2022

Reporting Summary

Nature Portfolio wishes to improve the reproducibility of the work that we publish. This form provides structure for consistency and transparency in reporting. For further information on Nature Portfolio policies, see our Editorial Policies and the Editorial Policy Checklist.

Statistics

For all statistical analyses, confirm that the following items are present in the figure legend, table legend, main text, or Methods section.

n/a	Confirmed	
☐	☒	The exact sample size (n) for each experimental group/condition, given as a discrete number and unit of measurement
☐	☒	A statement on whether measurements were taken from distinct samples or whether the same sample was measured repeatedly
☒	☐	The statistical test(s) used AND whether they are one- or two-sided<br>*Only common tests should be described solely by name; describe more complex techniques in the Methods section.*
☒	☐	A description of all covariates tested
☒	☐	A description of any assumptions or corrections, such as tests of normality and adjustment for multiple comparisons
☒	☐	A full description of the statistical parameters including central tendency (e.g. means) or other basic estimates (e.g. regression coefficient) AND variation (e.g. standard deviation) or associated estimates of uncertainty (e.g. confidence intervals)
☐	☒	For null hypothesis testing, the test statistic (e.g. F, t, r) with confidence intervals, effect sizes, degrees of freedom and P value noted<br>*Give P values as exact values whenever suitable.*
☒	☐	For Bayesian analysis, information on the choice of priors and Markov chain Monte Carlo settings
☒	☐	For hierarchical and complex designs, identification of the appropriate level for tests and full reporting of outcomes
☒	☐	Estimates of effect sizes (e.g. Cohen's d, Pearson's r), indicating how they were calculated

Our web collection on statistics for biologists contains articles on many of the points above.

Software and code

Policy information about availability of computer code

Data collection	no softwares were used
Data analysis	ImageJ V1.52g Prism 8, SPSS 13.0

For manuscripts utilizing custom algorithms or software that are central to the research but not yet described in published literature, software must be made available to editors and reviewers. We strongly encourage code deposition in a community repository (e.g. GitHub). See the Nature Portfolio guidelines for submitting code & software for further information.

Data

Policy information about availability of data

All manuscripts must include a data availability statement. This statement should provide the following information, where applicable:

- Accession codes, unique identifiers, or web links for publicly available datasets
- A description of any restrictions on data availability
- For clinical datasets or third party data, please ensure that the statement adheres to our policy

All relevant data are provided in this article and its supplementary information files, or are available from the corresponding authors upon reasonable request.

Field-specific reporting

Please select the one below that is the best fit for your research. If you are not sure, read the appropriate sections before making your selection.

☒ Life sciences ☐ Behavioural & social sciences ☐ Ecological, evolutionary & environmental sciences

For a reference copy of the document with all sections, see nature.com/documents/nr-reporting-summary-flat.pdf

Life sciences study design

All studies must disclose on these points even when the disclosure is negative.

Sample size	we chose the sample sizes≥4?We refer to many articles as reference for sample size
Data exclusions	No data is exclusion
Replication	All attempts at replication were successful
Randomization	the allocation was random
Blinding	the investigators were blinded to group allocation during data collection and analysis

Reporting for specific materials, systems and methods

We require information from authors about some types of materials, experimental systems and methods used in many studies. Here, indicate whether each material, system or method listed is relevant to your study. If you are not sure if a list item applies to your research, read the appropriate section before selecting a response.

Materials & experimental systems

n/a	Involved in the study	
☐	☒	Antibodies
☒	☐	Eukaryotic cell lines
☒	☐	Palaeontology and archaeology
☐	☒	Animals and other organisms
☒	☐	Human research participants
☒	☐	Clinical data
☒	☐	Dual use research of concern

Methods

n/a	Involved in the study	
☒	☐	ChIP-seq
☒	☐	Flow cytometry
☒	☐	MRI-based neuroimaging

Antibodies

Antibodies used	Two kinds of KDM3A primary antibodies were purchased from Abcam (Cambridge, UK) (ab106456) for western blotting assay and Santa Cruz (CA, USA) (sc-376608X) for ChIP-PCR detection, respectively. Primary antibodies used in the experiments are purchased from the following suppliers: H3K9me2 (ab1220), Bax (ab32503), Bcl-2 (ab196495), cleaved caspase-9 (ab52298), cleaved caspase-3 (ab2302), cytoplasmic cytochrome c (ab13575), mitochondrial cytochrome c (ab13575), VDAC1 (ab15895) and GAPDH (ab37168) were from Abcam; ETS1 was from Proteintech Group, Inc (12118-1 AP). The secondary antibodies, either HRP-linked goat-anti mouse IgG or goat anti-rabbit IgG was obtained from BIOSS (Beijing, China).
Validation	the validation of each primary antibody for the species is available in manufacture's website

Animals and other organisms

Policy information about studies involving animals; ARRIVE guidelines recommended for reporting animal research

Laboratory animals	All of the Sprague-Dawley (SD) rats ,male ,weighing 220-250 g were obtained from Vital River Laboratories (VRL, Beijing, China).
Wild animals	the study did not involve any wild animals
Field-collected samples	All of the Sprague-Dawley (SD) rats were bred in a standard environment with controlled temperature (20-25 °C), humidity (40-60%), light condition (12 h light/dark cycle), and with ad libitum provided food and water.
Ethics oversight	the study protocol was approved by the Institutional Animal Care and Use Committee from Wuhan University.

Note that full information on the approval of the study protocol must also be provided in the manuscript.
